# Clinical impact of EUCAST rapid antimicrobial susceptibility testing on empirical therapy in bloodstream infections: a real-world observational study

**DOI:** 10.1007/s15010-026-02829-4

**Published:** 2026-05-18

**Authors:** Lukas Schaffarczyk, Patricia Carla Mayer, Axel Hamprecht, Philipp Thelen

**Affiliations:** 1 Institute of Medical Microbiology and Virology, Carl von Ossietzky Universität Oldenburg, Oldenburg, Germany; 2 Institute of Medical Microbiology and Virology, Klinikum Oldenburg, Oldenburg, Germany

**Keywords:** Rapid antimicrobial susceptibility testing (RAST), Bloodstream infection, Antimicrobial stewardship, VITEK 2, Diagnostic turnaround time, Empirical therapy duration.frefere

## Abstract

**Purpose:**

This study assessed the impact of EUCAST’s Rapid Antimicrobial Susceptibility Testing (RAST) on empirical therapy and its clinical usability in routine bloodstream infection management.

**Methods:**

This retrospective single-center, observational study compared two four-month periods before and after RAST implementation (Feb–May 2023 vs. Feb–May 2024) at Oldenburg Medical Center. Patients with monomicrobial bloodstream infections due to *Escherichia coli*, *Klebsiella pneumoniae*, *Pseudomonas aeruginosa*, or *Acinetobacter baumannii* were included. Primary outcome: duration of empirical antimicrobial therapy. Secondary outcomes: turnaround time and categorical agreement between RAST with VITEK 2, including major errors (ME) and very major errors (VME). Surrogate-based interpretation was used for ATU results, particularly for piperacillin-tazobactam.

**Results:**

In total 126 patients were included (66 in 2023, 60 in 2024). After RAST, empirical therapy duration was significantly shorter (40.5 h vs. 73.3 h, *p* < 0.001). Median time to RAST result was 15.8 h, significantly shorter than by VITEK 2 (33.9 h, *p* < 0.0001), reducing time to first susceptibility result by 9.6 h (*p* < 0.00001). Concordance with VITEK 2 was excellent: 100% for cefotaxime, meropenem, and piperacillin-tazobactam, 96.4% for ceftazidime, 95.2% for ampicillin. ATU results occurred mainly for piperacillin-tazobactam and were partly resolved through surrogate-based interpretation.

**Conclusion:**

RAST significantly shortened empirical therapy duration and accelerated susceptibility reporting with high agreement to VITEK 2. However, antibiotic-specific uncertainty and incomplete translation of early results into therapeutic action indicate that the clinical impact of RAST depends on structured interpretation and integration into antimicrobial stewardship workflows.

## Introduction

Increasing antibiotic resistance poses a significant threat to global health [1]. One of the most effective strategies to counter this challenge is the timely identification of resistant pathogens, enabling the prompt initiation of targeted antimicrobial therapy. This is particularly critical in bloodstream infections, where delays in adequate treatment are associated with increased morbidity and mortality [2].

As the burden of infections caused by multidrug-resistant organisms continues to rise worldwide, the need for rapid and reliable diagnostic methods becomes increasingly urgent—especially in the context of sepsis. In such life-threatening infections, the time to appropriate antimicrobial therapy is a decisive factor in patient outcomes [3, 4]. Even in settings with a low prevalence of antimicrobial resistance, rapid and reliable susceptibility results might help shorten the duration of broad-spectrum antibiotic use and thereby support antimicrobial stewardship efforts.

Phenotypic antimicrobial susceptibility testing remains a cornerstone of resistance detection. To accelerate this process, the European Committee on Antimicrobial Susceptibility Testing (EUCAST) introduced Rapid Antimicrobial Susceptibility Testing (RAST) in April 2022 [5]. This method allows for direct susceptibility testing from positive blood cultures, with results available after only 4, 6, or 8 h and even 16–20 h of incubation using pathogen-specific breakpoints [6]. It is currently validated for *Escherichia coli*,* Klebsiella pneumoniae*,* Salmonella enterica*,* Pseudomonas aeruginosa*,* Staphylococcus aureus*,* Streptococcus pneumoniae*,* Enterococcus faecalis*,* Enterococcus faecium*, and *Acinetobacter baumannii* [7]. As a cost-effective and easily implementable technique, RAST holds significant potential to shorten diagnostic turnaround times in routine microbiological workflows—thereby contributing to faster clinical decision-making and improved patient outcomes.

Although several studies have demonstrated that RAST is a reliable diagnostic tool, its effect on the duration of empirical antimicrobial therapy remains insufficiently investigated [5, 8].

The aim of this study was (i) to analyze the impact of RAST on the duration of empirical antimicrobial therapy, specifically the time from initiation of treatment to the first adjustment prompted by susceptibility results. (ii) To evaluate the acceleration of diagnostic turnaround times by comparing the time from blood culture positivity to reporting of antimicrobial susceptibility results (time to first susceptibility result) before and after implementation at our hospital. (iii) To assess the accuracy of RAST compared to VITEK 2.

For the interpretation of piperacillin-tazobactam—a key empiric agent in Germany—we additionally applied a surrogate approach, inferring susceptibility from ampicillin and/or amoxicillin-clavulanic acid when results fell into the area of technical uncertainty (ATU) and ampicillin and/or amoxicillin-clavulanic were tested susceptible (S). This strategy was motivated by previous studies, which have shown that piperacillin-tazobactam frequently proved difficult to interpret in RAST and often resulted in zone diameters within the ATU [9, 10].

## Materials and methods

### Study design

This single-center, retrospective pre-post quasi-experimental observational study examines two time periods of 4 months each, before and after the implementation of RAST (February 1, 2023 - May 31, 2023 & February 1, 2024 - May 31, 2024). RAST was performed for the gram-negative pathogens *E. coli*, *P. aeruginosa*, *K. pneumoniae*, and *A. baumannii*. A prerequisite for RAST implementation was that blood cultures could be processed and reported during core working hours from 8 AM to 6 PM on Monday to Friday. Eligible samples included positive blood cultures whose positive signal was detected no more than 18 h prior. RAST readings were only taken at 6 h of incubation, as at this point most inhibitions zones can be read compared to 4 h of incubation [9, 11].

### Study population and setting

**Fig. 1:**
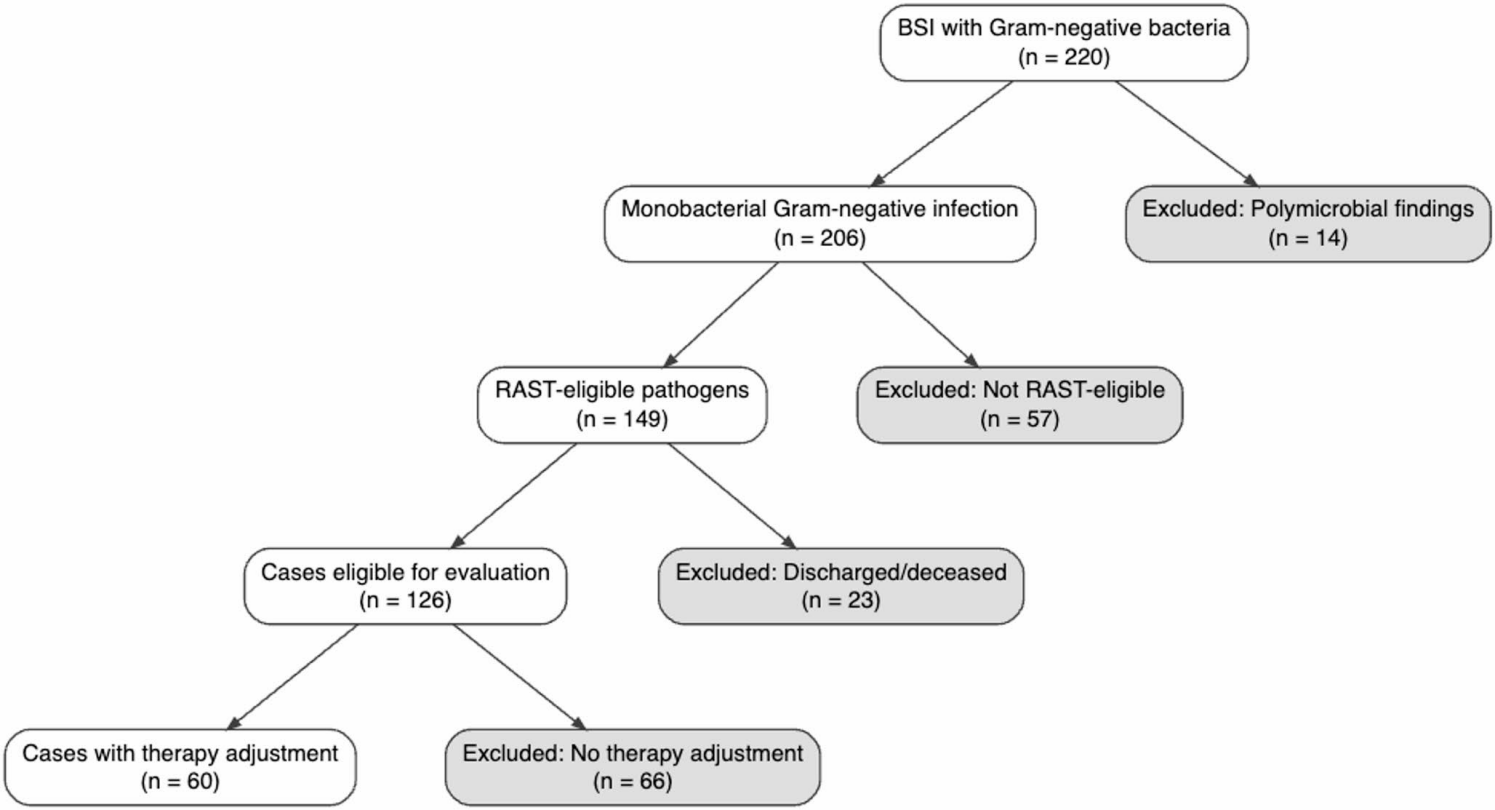
Inclusion and exclusion criteria for bloodstream infections (BSI)

The study population comprised patients diagnosed with bloodstream infections (BSI) caused by gram-negative pathogens at the Institute for Medical Microbiology and Virology in Oldenburg. The subjects were admitted to or received inpatient treatment at Oldenburg Medical Center during the periods of February 2023 to May 2023 and February 2024 to May 2024. Eligibility was limited to patients with monomicrobial bloodstream infections due to *Escherichia coli*, *Klebsiella pneumoniae*, *Pseudomonas aeruginosa*, or *Acinetobacter baumannii*, resulting in a final study cohort selected from the initial 149 cases (Fig. 1). Data collection was conducted using the laboratory and hospital information systems available at Oldenburg Medical Center (Medat LIS, Medat Computer-Systeme GmbH, Munich, Germany; Medico HIS, Cerner/Oracle, Germany; patient records). Pediatric blood culture bottles and those inoculated with other materials than blood (e.g. joint fluid) were excluded. Further exclusion criteria included patients with polymicrobial blood cultures containing gram-positive organisms, cases in which none of the aforementioned RAST-eligible species were isolated, and patients who had died, been discharged, or transferred before antimicrobial susceptibility testing results became available. In addition, cases without any antibiotic therapy modification in response to susceptibility results were excluded from the analysis assessing the impact of RAST on the duration of empirical therapy.

### Study protocol

#### Blood culture diagnostics

Blood culture bottles BACTEC™ PLUS AEROBIC and BACTEC™ LYTIC ANAEROBIC F-Medium (Beckton-Dickinson, USA) were incubated in a BD BACTEC™ FX blood culture system at a temperature of 37 °C. Positive blood cultures were removed from the device, and a Gram stain was prepared, the results of which were communicated by the microbiologist to the clinician via telephone.

#### Standard-method

From the positive blood culture bottle, with detection of gram-negative bacilli, one drop of blood (approximately 40 µl) was applied to a Columbia agar plate (BD, Heidelberg, Germany) and a chocolate agar plate (BD, Heidelberg, Germany) and incubated for 4 h at 37 °C in 5% CO₂. From this short-term culture, pathogen identification was performed using MALDI-TOF MS (Bruker Daltonics, Bremen, Germany). Additionally, a bacterial suspension with a turbidity equivalent to a 0.5 McFarland standard was prepared in saline solution, and subsequently, susceptibility testing was performed using the VITEK 2 automated susceptibility testing system with the AST-N223 card (bioMérieux, Nürtingen, Germany).

#### RAST-method

RAST-methodology was performed according to the EUCAST protocol [12]. Shortly, a Mueller-Hinton agar plate (Oxoid, Wesel, Germany) was inoculated with 125 ± 25 µL undiluted blood culture broth and evenly spread using a swab or plate rotator. Antibiotic disks (Oxoid, Wesel, Germany) are applied immediately, and plates are incubated at 35 ± 1 °C under aerobic conditions. The antibiotic panel included ampicillin 10 µg, amoxicillin-clavulanic acid 10–20 µg, piperacillin-tazobactam 30 –6 µg, cefotaxime 5 µg, ceftazidime 10 µg, meropenem 10 µg. After 6 h (± 5 min), inhibition zones were evaluated only if bacterial growth is confluent and zone edges are clearly defined. Results were interpreted according to the breakpoints for Rapid AST directly from positive blood culture bottles (Version 7.0). In several cases, antimicrobial susceptibility testing for piperacillin-tazobactam yielded inhibition zone diameters within the Area of Technical Uncertainty (ATU), as defined by EUCAST. Deviating from the original protocol, the interpretation of amoxicillin-clavulanic acid and ampicillin susceptibility was assessed as a surrogate marker in such cases. This approach was based on a retrospective analysis of 10,835 AST results from *E. coli* and *K. pneumoniae* isolates performed with VITEK 2 between 2020 and 2023 in our institute, in which the amoxicillin-clavulanic acid MIC was ≤ 8 mg/L (susceptible category). Among these, only 72 isolates (0.007%) showed a piperacillin-tazobactam MIC within the ATU range, and 14 isolates (0.002%) were resistant to piperacillin-tazobactam. Given the rarity of discordant results, susceptibility to piperacillin-tazobactam was inferred when susceptibility to either surrogate agent was confirmed. Conversely, no inference was made in cases of resistance or when surrogate results themselves fell within the ATU range. Surrogate-based inference was thus deliberately restricted to susceptible categorizations.

#### Time from positive blood culture to susceptibility result

To compare the time to susceptibility results between 2023 and 2024, we extracted time difference (in hours) from first positive blood culture signal to (i) VITEK 2 result, (ii) RAST result, and (iii) the first susceptibility result (either VITEK 2 or RAST). For each of these time points, we assessed the distribution using the Shapiro–Wilk normality test. Since none of the groups showed a normal distribution (all *p* < 0.05), we reported medians and interquartile ranges (IQRs) and used the Wilcoxon rank-sum test (Mann–Whitney U test) for group comparisons.

#### Duration and adjustment of empirical antimicrobial therapy

The duration of empirical antimicrobial therapy was defined as the time (in hours) from the initial administration of empirical treatment to the first adjustment based on susceptibility test results. Time differences were calculated using timestamps for therapy initiation and documented therapy changes.

Patients were grouped by study year and diagnostic method: *2023 (VITEK 2 only)*, *2024 (all cases)*, *2024 (VITEK 2 only)*, *2024 (RAST only).* For each group, we calculated the mean, standard deviation (SD) and range. Data distribution was assessed using the Shapiro-Wilk test. Depending on the normality of data, comparisons between groups were performed using either Welch’s t-test or the Wilcoxon rank-sum test. A p-value < 0.05 was considered statistically significant.

To further evaluate the impact of RAST on the adjustment of empirical therapy, we classified whether escalation or de-escalation of the current antimicrobial regimen was deemed necessary based on the results of RAST.

De-escalation was defined as the switch from a broad-spectrum regimen (primarily piperacillin-tazobactam or meropenem) to narrower-spectrum agents such as aminopenicillins (with or without β-lactamase inhibitor) or the discontinuation of combination partners, provided targeted activity was maintained.

Escalation was defined as the addition of further agents or a switch to broader-spectrum antibiotics (e.g., escalation to carbapenems). The classification was performed by the study team and members of the local antimicrobial stewardship team.

Cases without any antibiotic therapy modification in response to susceptibility results were excluded from this analysis.

#### Analysis of susceptibility test results

For data analysis, we used the terms categorical agreement, very major errors, major errors, and minor errors. A very major error (VME) was defined as a susceptible result from rapid antimicrobial susceptibility testing (RAST), while the standard method, VITEK 2, indicated resistance. A major error (ME) occurred when RAST-method reported resistance, but VITEK 2 showed susceptibility. To assess the categorical agreement between RAST results and the reference method (VITEK 2), we calculated the prevalence-adjusted bias-adjusted kappa (PABAK) for each antibiotic tested. The PABAK was chosen over Cohen’s kappa to account for possible distortions due to high prevalence or systematic bias in sensitivity or resistance rates. PABAK was calculated as 2*P*_o_​−1, where *P*_o_​ represents the observed agreement between RAST and VITEK 2 results. Only isolate-antibiotic combinations where RAST was performed and both methods yielded interpretable categorical results (susceptible or resistant) were included in the analysis. Since AMC is not included on the routinely used VITEK 2 card at our institution (as AMC is not applied as intravenous therapy in Germany), it was not included in patient reports and was used exclusively to infer piperacillin-tazobactam susceptibility when tested susceptible.

Measurements that fell within the EUCAST-defined Area of Technical Uncertainty (ATU) were excluded from the agreement analysis to avoid classification bias. The PABAK values were interpreted as follows: values ≥ 0.81 indicated very good agreement, 0.61–0.80 good, 0.41–0.60 moderate, 0.21–0.40 fair, 0.00–0.20 slight, and values < 0 indicated agreement less than expected by chance (Table 5).

For the evaluation of test performance, all isolates subjected to RAST were included in the analysis, regardless of any resulting changes in antimicrobial therapy.

### Statistical analysis

All analyses were performed using RStudio (version 2025.05.0 + 496).

## Results

### Study population

The study population included 126 patients with bloodstream infections caused by one of the four investigated pathogens (*E. coli*,* K. pneumoniae*,* P. aeruginosa or A. baumannii*), documented in 2023 (*n* = 66) and 2024 (*n* = 60) during the period from February 1 to May 31 (Fig. 1). Data were stratified by gender, age group, and clinical department. Overall, 60.8% of patients were male. Patients aged 61–80 years constituted the largest age group (36.0%), while 22.4% were older than 80 years. Most infections occurred in patients from the urology department (32.0%), followed by oncology (23.2%) and gastroenterology (12.8%).

Inclusion and exclusion criteria for bloodstream infections (BSI) caused by Gram-negative bacteria between February and May of 2023 and 2024. Only monomicrobial cases with pathogens eligible for rapid antimicrobial susceptibility testing (RAST) and a subsequent antibiotic therapy adjustment were included in the final analysis.

### Pathogen distribution

The distribution among RAST-eligible pathogens was similar in both study years. *Escherichia coli* was the most frequently isolated species, accounting for 76.8% of all cases (74.2% in 2023 and 79.7% in 2024). *Klebsiella pneumoniae* was identified in 14.4% of patients, with comparable proportions in both years (13.6% in 2023 and 15.3% in 2024). *Pseudomonas aeruginosa* was less common, detected in 9.5% of cases overall, with a higher proportion in 2023 (12.1%) compared to 2024 (6.8%). No cases involving *Acinetobacter baumannii* were observed in either year (Table 1).

**Table 1:** Distribution of RAST-Eligible Pathogens

Pathogen	2023 (*n* = 66)	2024 (*n* = 60)	Total (*n* = 126)
*Escherichia coli*	49 (74.2%)	47 (78.3%)	96 (76.2%)
*Klebsiella pneumoniae*	9 (13.6%)	9 (15.0%)	18 (14.3%)
*Pseudomonas aeruginosa*	8 (12.1%)	4 (6.7%)	12 (9.5%)
*Acinetobacter baumannii*	0 (0.0%)	0 (0.0%)	0 (0.0%)

Data are presented as absolute numbers and percentages. Percentages refer to the respective year’s total (2023: *n* = 66, 2024: *n* = 60, Total: *n* = 126).

Overall, resistance rates remained low for broad-spectrum β-lactams and unchanged across study years. *E. coli* showed the highest resistance to ampicillin (≈ 50%) and ampicillin-sulbactam (≈ 50%), with moderate rates for piperacillin-tazobactam (6–17%), and low resistance to third-generation cephalosporins (≤ 14%). *K. pneumoniae* exhibited intrinsic ampicillin resistance and moderate resistance to β-lactam/β-lactamase-inhibitor combinations (≤ 33%), while *P. aeruginosa* remained largely susceptible, with only low resistance to piperacillin-tazobactam and ceftazidime. All isolates were susceptible to meropenem (Table 6).

In 2024, a total of 30 RAST analyses were performed during core working hours, which accounts for 50% of all eligible cases (*n* = 60).

**Table 6:** Antimicrobial resistance rates of RAST-eligible Gram-negative pathogens in 2023 and 2024 (VITEK 2)

Pathogene	number	Year	AMP	ASU	TZP	CTX	CAZ	MER
*E coli*	49	2023	46.9	46.9	6.1	6.1	6.1	0
*E coli*	47	2024	53.8	51.1	17	13.5	12.6	0
*K. pneumoniae*	9	2023	-	22.2	11.1	0	0	0
*K. pneumoniae*	9	2024	-	33.3	22.2	0	0	0
*P. aeruginosa*	8	2023	-	-	12.5	-	0	0
*P. aeruginosa*	4	2024	-	-	25	-	12.5	0

AMP, ampicillin; ASU, ampicillin-sulbactam; TZP, piperacillin-tazobactam; CTX, cefotaxime; CAZ, ceftazidime; MER, meropenem. Values indicate the proportion of resistant isolates (%). “–” indicates that the antibiotic was not tested for the respective species due to intrinsic resistance.

### Performance of RAST compared to VITEK 2

Between 28 and 30 RAST results per antibiotic were available for comparison with VITEK 2. The number of tested antibiotics varied across isolates, owing to the intrinsic resistance of *Pseudomonas aeruginosa* to ampicillin and cefotaxime, for which no EUCAST breakpoints are defined. Accordingly, 28 isolates were tested for ampicillin and cefotaxime, and 30 each for ceftazidime, meropenem, and piperacillin-tazobactam. Fourteen piperacillin-tazobactam results were excluded because they fell within the area of technical uncertainty (ATU). In addition, one result for each substance (3.3–4.3%) was not readable at 6 h of incubation (Table 5).

**Table 5:** Categorical agreement and PABAK between RAST and VITEK 2

	AMP	CTX	CAZ	MER	TZP
	All RAST cases				
Tests performed	28	28	30	30	30
Susceptible	10 (37%)	23 (82.1%)	23 (76.7%)	29 (96.7%)	10 (33.3%)
Susceptible with increased exposure	0	0	2 (6.7%)	0	2
Resistant	16 (59.3%)	4 (14.3%)	3 (10%)	0	3 (10%)
ATU	0	0	1 (3.3%)	0	14 (46.7%)
Not assessable	1 (3.6%)	1 (3.6%)	1 (3.3%)	1 (3.3%)	1 (3.3%)
PABAK-Index	1.000	1.000	0.923	1.000	1.000
	Categorical Agreement (compared to VITEK 2)				
Total assessable	27	27	28	29	15
cA	26 (96.3%)	27 (100%)	27 (96.6%)	29 (100%)	15 (100%)
ME	0	0	1 (3.4%)	0	0
VME	1 (3.7%)	0	0	0	0

AMP = ampicillin, CTX = cefotaxime, CAZ = ceftazidime, MER = meropenem, TZP = piperacillin-tazobactam, RAST = rapid antimicrobial susceptibility testing, ATU = area of technical uncertainty, PABAK = prevalence-adjusted bias-adjusted kappa, cA = categorical agreement, ME = major error, VME = very major error; For piperacillin-tazobactam (TZP), 14 results fell into the ATU range. In 5 of these cases, susceptibility could be inferred using surrogate markers (AMP/AMC), which was in complete concordance (100%) with VITEK 2.

The resulting number of assessable cases was 27 for ampicillin, 27 for cefotaxime, 28 for ceftazidime, 29 for meropenem, and 15 for piperacillin-tazobactam. Categorical agreement (CA) with VITEK 2 was highest for cefotaxime, meropenem, and piperacillin-tazobactam (100%), followed by ceftazidime (96.6%) and ampicillin (95.5%). One major error occurred for ceftazidime, while one very major error was detected for ampicillin. No errors were recorded for cefotaxime, meropenem, or piperacillin-tazobactam.

A total of 14 isolates showed piperacillin-tazobactam (TZP) results within the area of technical uncertainty (ATU). In these cases, surrogate-based interpretation using amoxicillin-clavulanic acid (AMC) was assessed and enabled classification as susceptible in 5/14 isolates.

In 5/14 cases, surrogate-based interpretation using AMC was not possible because AMC itself fell within the ATU range. In one of these cases, both AMC and TZP were within the ATU. However, susceptibility could be inferred based on ampicillin, which was susceptible.

In the remaining 4/14 cases, TZP was initially reported as resistant. VITEK 2 result identified these isolates as susceptible.

Among isolates with AMC results outside the ATU (*n* = 8), all were concordant with TZP susceptibility. Notably, in all cases where ampicillin was categorized as susceptible, this was concordant with susceptibility to both AMC and piperacillin-tazobactam.

Prevalence-adjusted bias-adjusted kappa (PABAK) values confirmed high agreement (≥ 0.909) for all tested β-lactams.

### Diagnostic turnaround time

The median time from blood culture positivity to VITEK 2 result in 2023 was 33.87 h (IQR 29.27–42.22). In 2024, the median was 34.03 h (IQR 28.97–39.05), with no statistically significant difference (*p* = 0.72).

For RAST results in 2024, the median time was significantly shorter at 15.75 h (IQR 11.7–17.4) compared to 33.87 h for VITEK results in 2023 (*p* < 0.00001).

Similarly, the time to the first susceptibility result (TTFS) was also significantly reduced in 2024 with a median of 24.25 h (IQR 16.1–36.43), corresponding to a median reduction of 9.62 h (*p* < 0.00001) (Table 2).

**Table 2:** Time to Diagnostic Results

	2023 (VITEK) median	2024 (VITEK) median	2024 (RAST) median	2024 (first result: RAST or VITEK) median
=Time to VITEK result (TTV)=	33.87 h (IQR 29.27–42.22)	34.03 h (IQR 28.97–39.05)	–	–
Time to RAST result (TTR)	–	–	15.75 h (IQR 11.7–17.4)	–
Time to first susceptibility result (TTFS)	–	–	–	24.25 h (IQR 16.1–36.43h)
Time saved 2023 (VITEK) vs. 2024 (RAST & VITEK)	–	–	-18.12 h (*p* < 0.00001)	−9.62 h (*p* < 0.00001)

Time (in hours) from blood culture positivity to availability of susceptibility results by method. TTV = Time to VITEK result; TTR = Time to RAST result; TTFS = Time to first susceptibility result (either RAST or VITEK). SD = standard deviation. Values are reported as medians with interquartile ranges (IQR). Statistical significance was assessed using the Wilcoxon rank-sum test due to non-normal data distribution (Shapiro–Wilk test).

### Empiric antimicrobial therapy across study groups

**Fig. 2:**
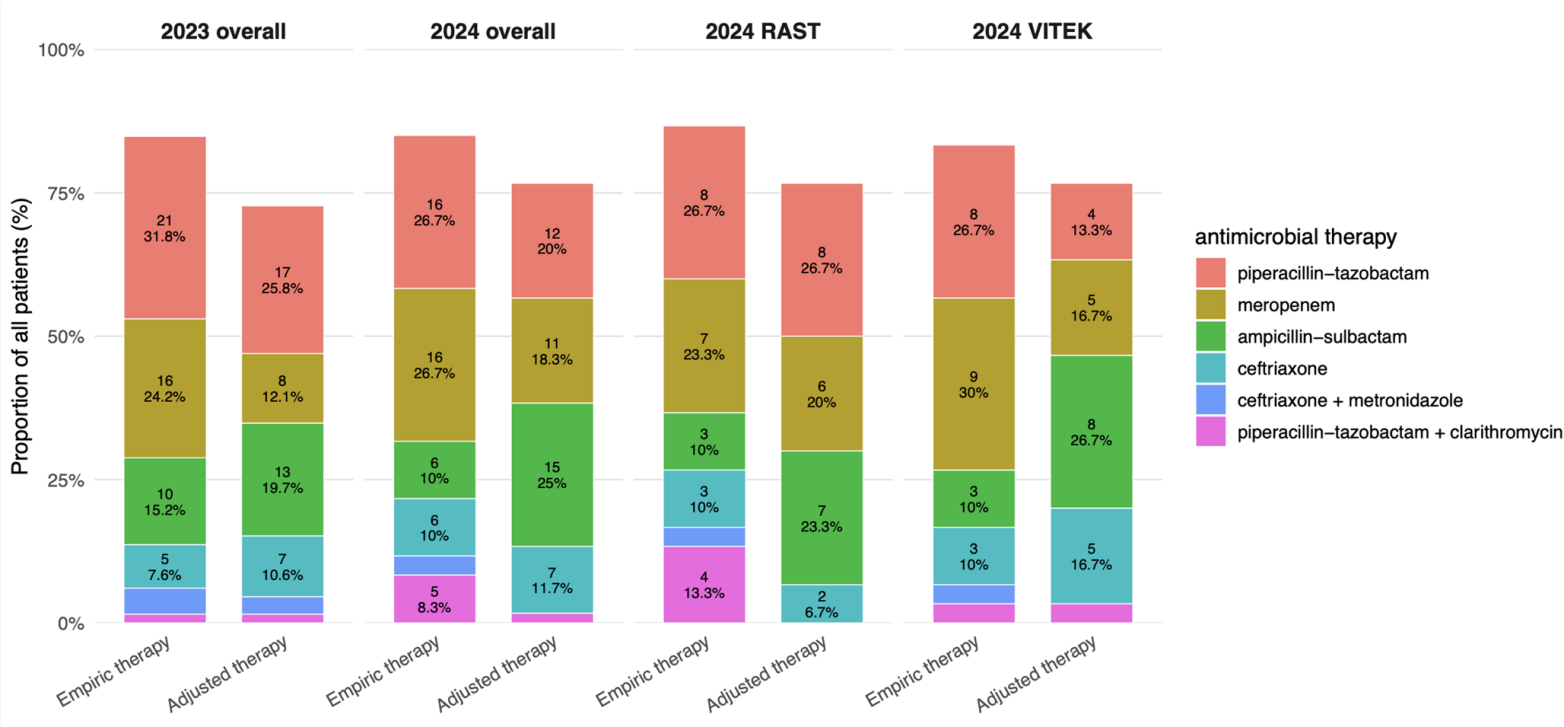
Comparison of empiric and adjusted antimicrobial therapy

The empiric antibiotic therapy regimen changed only minimally between 2023 and 2024. Piperacillin-tazobactam was more frequently used in 2023 (31.8%), but the usage rates of both meropenem and piperacillin-tazobactam were identical in 2024 (both 26.7%). In 2024 the use of empiric therapy regimens varied slightly between subgroups, with no clear standout agent emerging as the most frequently used treatment option (Fig. 2).

The empirical antimicrobial therapy administered in the RAST group was almost entirely covered by the RAST panel (meropenem, piperacillin-tazobactam, ampicillin-sulbactam, and ceftriaxone), with cefazolin as the only exception.

Stacked bar charts comparing empiric therapy at the time of blood culture collection and adjusted therapy after availability of antimicrobial susceptibility results. Cases without therapy modification were classified as unchanged. Only selected regimens are displayed. Percentages are calculated based on all patients within each group and timepoint.

### Impact of RAST results on antimicrobial therapy adjustments

RAST results did not allow interpretation of the ongoing empirical antibiotic therapy in eight out of 30 patients in the RAST group. In most of these (*n* = 4/8), this was due to piperacillin-tazobactam (TZP) being within the ATU range. In one of these patients, therapy was switched to meropenem, as no RAST result for TZP was available. Three patients received ampicillin-sulbactam (ASU), which could not be inferred from the RAST result since only ampicillin was tested (*n* = 3/8), and no RAST breakpoints are available for ampicillin-sulbactam yet. In one additional case, the RAST result was unreadable.

Among the remaining 22 patients, de-escalation of antimicrobial therapy based on RAST would have been possible in 59.1% (13/22), but same-day de-escalation was implemented in only 30.8% of these cases (*n* = 4/13).

Escalation of antimicrobial therapy was indicated in 13.6% (*n* = 3/22) and was performed on the same day in 66.7% of those cases (2/3) (Table 3).

**Table 3:** Impact of RAST results on same-day antimicrobial therapy adjustments (2024)

Parameter	2024 (RAST)
No therapeutic interpretation possible	8 / 30 (26.7%)
De-escalation possible	13 / 22 (59.1%)
De-escalation performed (same day)	4 / 13 (30.8%)
Escalation necessary	3 / 22 (13.6%)
Escalation performed (same day)	2 / 3 (66.7%)
Therapy modification (same day, prompted by RAST)	6 / 16 (37.5%)

Proportion of cases with potential or actual antimicrobial de-escalation or escalation on the day of RAST result availability. The category “No therapeutic interpretation possible” includes RAST results that were technically evaluable but did not allow a clinical assessment of de- or escalation (e.g., key antibiotics within the ATU range). Only interpretable RAST results (*n* = 22) were included in the subsequent analyses. One additional case (*n* = 1) showed a same-day therapy change, but the necessity was not classifiable as de- or escalation after RAST.

Overall, RAST results led to a same-day therapy modification in (6/16) of cases in which RAST provided an option for escalation or de-escalation. In one case, a necessary escalation was not recognized due to a very major error (VME) in the RAST result. Ampicillin was tested susceptible by RAST which turned out to be resistant by VITEK 2 and MIC Strip.

### Duration of Empirical Antimicrobial Therapy

In 2023, when only VITEK 2 was used, the duration of empirical antimicrobial therapy averaged 73.3 h (SD 28.9; range 27.8–130.4 h). In 2024, the mean duration was significantly shorter in the overall cohort (VITEK 2 + RAST; 53.5 h, SD 27.8; range 7.3–134.6 h; *p* = 0.013 vs. 2023). Within 2024 subgroups, patients tested by VITEK 2 only had a comparable mean therapy duration (70.2 h, SD 28.5; range 40.4–134.6 h; *p* = 0.77 vs. 2023), while those tested by RAST alone showed a markedly shorter mean therapy duration (40.5 h, SD 19.5; range 7.3–72.0 h; *p* < 0.001 vs. 2023) (Table 4).

**Table 4:** Duration of Empirical Antimicrobial Therapy

	Mean (h)	SD	Range (h)	*p*-value
2023 (VITEK only) (*n* = 26)	73.3	28.9	27.8–130.4	–
2024 (VITEK + RAST) (*n* = 32)	53.5	27.8	7.32–134.6	0.01380
2024 (VITEK only) (*n* = 14)	70.2	28.5	40.35–134.6	0.76590
2024 (RAST only) (*n* = 18)	40.5	19.5	7.32–72.0	< 0.001

Duration of empirical therapy (in hours) from initiation to adaptation based on susceptibility results. Comparison across study years and diagnostic approaches. SD = standard deviation. All p-values refer to comparisons with the 2023 (VITEK only) group.

## Discussion

The present study evaluates the clinical impact of EUCAST rapid antimicrobial susceptibility testing (RAST) beyond analytical performance, with a particular focus on its influence on empirical antibiotic therapy and its usability under routine clinical conditions. While rapid diagnostics are often assumed to directly translate into earlier therapeutic action, real-world data on how early susceptibility results are interpreted and acted upon in daily practice remain limited. By combining diagnostic turnaround times, agreement analyses, and therapy modification data, our study provides insight into both the benefits and the practical constraints of implementing RAST in a routine hospital setting.

The introduction of EUCAST rapid antimicrobial susceptibility testing (RAST) led to a marked reduction in duration of empirical antimicrobial therapy and demonstrating excellent categorical agreement with VITEK 2. For key β-lactam antibiotics such as cefotaxime, meropenem, and piperacillin-tazobactam, agreement reached 100% and only isolated discrepancies occurred for ampicillin and ceftazidime. These findings support the analytical reliability of RAST for a broad range of clinically relevant agents [9]. A particular challenge arose with piperacillin-tazobactam, where a considerable proportion of results fell within the area of technical uncertainty (ATU), a phenomenon that has been described in previous studies [7, 9, 13]. As piperacillin-tazobactam is one of the most widely used agents for empiric antimicrobial therapy in Germany, such non-reportable results may drive unintended escalation of antibiotic therapy, thereby limiting the immediate clinical usefulness of otherwise rapid susceptibility results [9, 14].

By applying a surrogate approach using amoxicillin-clavulanic acid or ampicillin, more than one third of ATU results could be classified as susceptible, all in full agreement with the final AST result. This suggests that carefully defined surrogate interpretation may enhance the clinical utility of RAST without compromising accuracy and help to avoid unnecessary escalation of therapy.

Surrogate-based interpretation was applied prospectively and deliberately restricted to susceptible categorizations, reflecting a safety-oriented strategy aligned with antimicrobial stewardship principles. However, applicability was limited, as amoxicillin-clavulanic acid frequently fell within the ATU. Notably, in one case where both amoxicillin-clavulanic acid and piperacillin-tazobactam were within the ATU, susceptibility could be inferred based on ampicillin, which was consistently concordant with both agents in all evaluable cases.

This strategy was primarily informed by the retrospective analysis of our local antimicrobial resistance surveillance, rather than by microbiological rationale alone. While no common resistance mechanism in Enterobacterales is known to reliably produce susceptibility to amoxicillin-clavulanic acid alongside resistance to piperacillin-tazobactam, exceptions exist. For instance, certain beta-lactamases such as OXA-1 can result in a phenotype where the MIC of amoxicillin-clavulanic acid is lower than that of piperacillin-tazobactam, though typically no longer within the wild-type distribution [21]. Therefore, the use of surrogate markers is dependent on local epidemiology and cannot be generalized. It requires case-by-case evaluation in the context of local resistance patterns.

The analysis of time to result confirmed the diagnostic advantage of the method. While turnaround for VITEK 2 alone remained unchanged across study periods, indicating consistent laboratory processing, RAST enabled results to be reported after a median of about 16 h. This led to a median reduction of approximately 10 h in the time to first susceptibility result (TTFS), defined as the earliest available result from either VITEK or RAST, compared to the previous year, despite RAST being performed in only about half of the eligible cases due to limited processing and reporting hours. This demonstrates that meaningful diagnostic acceleration can be realized even without continuous 24/7 operation, provided that early results are timely communicated and interpreted within existing clinical workflows.

The impact of RAST results on therapeutic decisions was more nuanced. Although de-escalation was considered feasible in a large proportion of interpretable cases, same-day implementation occurred less frequently, indicating that availability of early susceptibility data alone does not guarantee therapeutic action. In approximately one quarter of all RAST analyses, a therapeutic interpretation was not possible. In most of these cases, the relevant agent — piperacillin-tazobactam — fell within the ATU and could not be inferred from ampicillin or amoxicillin-clavulanic acid. Consequently, definite susceptibility interpretation could be provided, and these results did not support direct therapeutic decisions.

Beyond this, the low uptake of de-escalation after RAST may reflect limited user experience with the method, the timing of result reporting, or organizational factors such as reduced availability of senior physicians at the end of the working day. The patient population may have played an additional role: roughly one quarter of cases originated from oncology or intensive care units, where de-escalation is often deferred because of patient complexity and perceived infection risk [15]. These findings underline that earlier result availability and additional reporting via phone alone does not automatically translate into clinical action unless embedded within established workflows and supported by antimicrobial stewardship structures. Escalation of therapy was considered necessary in a relatively few cases reflecting the low prevalence of multidrug-resistant Gram-negative organisms in our institution. Compared to de-escalation, therapy escalation was deemed necessary in three cases and implemented in two of them. In the single case without escalation, piperacillin-tazobactam tested within the ATU range and third-generation cephalosporins were resistant, suggesting ESBL production. Escalation was recommended but not performed [16–19]. Given the small number of cases, no reliable trend regarding decision-making behavior can be inferred. Nevertheless, this observation highlights the need for cautious interpretation of early susceptibility results, particularly in settings with low resistance prevalence.

The overall duration of empirical therapy was significantly shorter in patients tested by RAST, with reductions exceeding 30 h compared to the previous year’s VITEK cohort. This effect persisted when all 2024 cases were analyzed irrespective of testing method. The magnitude of this reduction should, however, be interpreted with caution, as only patients who underwent antibiotic modification were included. In roughly half of all cases, empirical therapy was continued unchanged, even though two-thirds of these patients were eligible for a narrower regimen. Despite the limited sample size, the observed difference remained statistically significant, underscoring the robustness of the finding. Previous studies have assessed therapy modifications following RAST [14, 20], but data on its quantitative impact on duration of empirical antimicrobial treatment are lacking. Our findings therefore add real-world evidence not only on diagnostic acceleration but also on the practical therapeutic relevance of RAST in routine care.

The present study therefore adds real-world evidence regarding both diagnostic acceleration and therapeutic relevance, of RAST in routine practice, particularly regarding the duration of empirical therapy.

Overall, these results indicate that RAST offers tangible benefits by reducing broad-spectrum antibiotic exposure and enabling earlier initiation of targeted therapy. Nevertheless, the observation that more than half of patients eligible for de-escalation remained on empirical regimens highlights the need for targeted antimicrobial stewardship interventions that emphasize the safety of narrowing therapy and foster direct communication between stewardship teams and treating clinicians.

Several limitations must be acknowledged. This was a single-center study with a moderate sample size, and RAST could be performed only in about half of the eligible cases due to restricted operating hours, introducing potential selection bias. In addition, VITEK 2 was used as the reference method rather than broth microdilution, which represents the gold standard for susceptibility testing. This approach was chosen for reasons of practicability within the routine diagnostic workflow. The low prevalence of multidrug-resistant isolates may have further limited the measurable impact of rapid resistance detection. Finally, the retrospective design and reliance on documentation of therapy changes do not allow for causal inference regarding clinical decision-making.

In summary, RAST proved to be a reliable and clinically valuable tool that reduces time to result and shortens the duration of empirical therapy under routine conditions. However, our findings demonstrate that diagnostic speed alone is insufficient to ensure timely therapeutic action. The clinical benefit of RAST is critically dependent on structured interpretation, clinician confidence, and integration into established antimicrobial stewardship workflows. Only when rapid susceptibility results are actively embedded into decision-making processes can their full potential to reduce unnecessary broad-spectrum antibiotic exposure be realized.

## Data Availability

The datasets generated during and/or analyzed during the current study are available from the corresponding author on reasonable request.
